# Narrow-bandwidth sensing of high-frequency fields with continuous dynamical decoupling

**DOI:** 10.1038/s41467-017-01159-2

**Published:** 2017-10-19

**Authors:** Alexander Stark, Nati Aharon, Thomas Unden, Daniel Louzon, Alexander Huck, Alex Retzker, Ulrik L. Andersen, Fedor Jelezko

**Affiliations:** 1 Department of Physics, Technical University of Denmark, Fysikvej, Kongens Lyngby, 2800 Denmark; 2 Institute for Quantum Optics, Ulm University, Albert-Einstein-Allee 11, Ulm, 89081 Germany; 3 Racah Institute of Physics, The Hebrew University of Jerusalem, Jerusalem, 91904 Israel; 4 Center for Integrated Quantum Science and Technology (IQst), Ulm University, Ulm, 89081 Germany

## Abstract

State-of-the-art methods for sensing weak AC fields are only efficient in the low frequency domain (<10 MHz). The inefficiency of sensing high-frequency signals is due to the lack of ability to use dynamical decoupling. In this paper we show that dynamical decoupling can be incorporated into high-frequency sensing schemes and by this we demonstrate that the high sensitivity achieved for low frequency can be extended to the whole spectrum. While our scheme is general and suitable to a variety of atomic and solid-state systems, we experimentally demonstrate it with the nitrogen-vacancy center in diamond. For a diamond with natural abundance of ^13^C, we achieve coherence times up to 1.43 ms resulting in a smallest detectable magnetic field strength of 4 nT at 1.6 GHz. Attributed to the inherent nature of our scheme, we observe an additional increase in coherence time due to the signal itself.

## Abstract (web summary)

State-of-the-art methods for sensing weak AC fields are only efficient in the low frequency domain. Here, Stark et al. demonstrate a sensing scheme that is capable of probing high frequencies through continuous dynamical coupling by applying it to a nitrogen-vacancy centre in diamond.

## Introduction

Improving the sensitivity of high-frequency sensing schemes is of great significance, especially for classical fields sensing^1–3^, detection of electron spins in solids^4, 5^, and nuclear magnetic resonance spectroscopy^6^. The common method to detect high-frequency field components is based on relaxation measurements, where the signal induces an observable effect on the lifetime, *T*_1_, of the probe system^4, 5, 7^. Nevertheless, the sensitivity of this method is limited by the pure dephasing time $T_2^*$ of the probe system.

Pulsed dynamical decoupling^8–10^ can substantially increase the coherence time^11–18^. In order to carry out sensing with a decoupling scheme, the frequency of the decoupling pulses has to be matched with the frequency of the target field^19, 20^. This largely restricts the approach to low frequencies, as the repetitive application of pulses is limited by the maximum available power per pulse^21^. The same power restrictions are present for very rapid and composite pulse sequences aimed to decrease both external and controller noise^22–26^.

With continuous dynamical decoupling (CDD)^21, 27–38^ robustness to external and controller noise can be attained, especially for multi-level systems^39–42^. However, the significance of CDD for sensing high-frequency fields remained elusive. Indeed, it was unclear whether it is possible to incorporate such a protection into the metrology task of sensing frequencies in the GHz domain. The first step towards this goal was done recently by integrating CDD in the sensing of high-frequency fields with three level systems^42^.

In this article, we propose, analyze, and experimentally demonstrate a sensing scheme that is capable of probing high-frequency signals with a coherence time, *T*_2_, limited sensitivity. Unlike relaxation measurements comprising a bandwidth ∝$1{\rm{/}}T_2^*$, determined by the pure dephasing time, $T_2^*$, of the sensor (up to the MHz range), our protocol overcomes the imposed limitation by protecting the addressed two-level system (TLS) with an adapted concatenated CDD approach. We use and adjust it such that high-frequency sensing becomes feasible even for not phase-matched signals. As a result, the proposed scheme is generic and works for many atomic or solid-state TLS, in which the energy gap matches the frequency of the signal under interrogation. A remarkable feature of our scheme is the fact that the signal to be probed also works partially as a decoupling drive and thus improves further the sensitivity of the sensor.

We demonstrate the performance of CDD by applying it to a nitrogen-vacancy (NV) center in diamond with natural abundance of ^13^C. Here, we utilize two of its ground sub-levels as the TLS. The states of the NV center can be read out and initialized by a 532 nm laser, which reveals spin-dependent fluorescence between the two levels^43–45^. The system can be manipulated by driving it with microwave fields. We show that by using a concatenation of two drives, an improvement in coherence time of the sensor by more than one order of magnitude is achieved. Taking into account the effect of an external signal, *g*, on the sensor during a concatenation of two drive fields, we obtain an improvement in bandwidth for high-frequency sensing by three orders of magnitude in comparison to the relaxometry approach. Moreover, we report on the measurement of a weak high-frequency signal with strength *g*, which relates to a smallest detectable magnetic field amplitude of δ*B*_min_ ≈ 4 nT.

## Results

### The sensing scheme

**Fig. 1:**
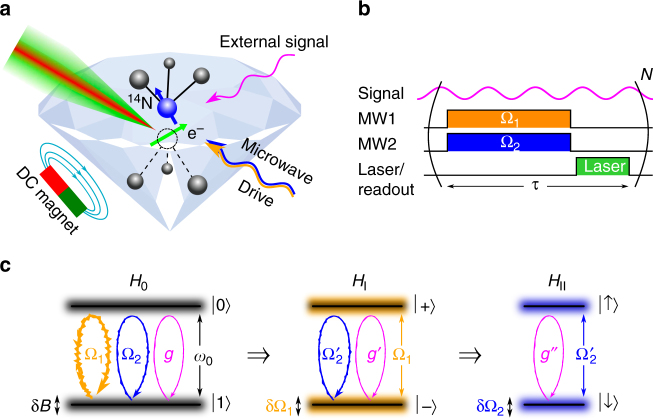
Schematic representation of our setup. **a** The NV center probes an external signal while it is being manipulated by the control fields. **b** Schematic representation of the sequence applied in this work. **c** The protected TLS: the bare system, *H*_0_, is subjected to strong environmental noise δ*B*. Applying a strong drive, Ω_1_, opens a protected gap, now subjected mainly to drive fluctuations δΩ_1_. A second drive, Ω_2_, is then applied to protect the TLS, *H*_*I*_, from these fluctuations, resulting in a TLS, *H*_*II*_, on resonance with the signal, *g*″ = *g*/4, with noise mainly from the second weak drive ${\rm{\delta }}{{\rm{\Omega }}_2} \ll {\rm{\delta }}{{\rm{\Omega }}_1}$

The basic idea of utilizing concatenated continuous driving to create a robust qubit is illustrated in Fig. 1a, b. The concatenation of two phase-matched driving fields results in a robust qubit^36, 42^. In what follows we show that such a robust qubit can be utilized as a sensor for frequencies in the range of the qubit’s energy separation and hence, dynamical decoupling can be integrated into the sensing task.

By the concatenated driving, the qubit is prepared in a state that allows for strong coherent coupling to the high-frequency signal to be probed (corresponding to the last TLS in Fig. 1c). In the total Hamiltonian, *H*, we consider the concatenation of two driving fields of strength (the Rabi frequency) Ω_1_ and Ω_2_, respectively. The Hamiltonians of the TLS, *H*_0_, the protecting driving fields, ${H_{{{\rm{\Omega }}_1}}},{H_{{{\rm{\Omega }}_2}}}$ and the signal, *H*_*s*_, are given by

1 $$\begin{array}{*{20}{l}}\\ H 	= {H_0} + {H_{{{\rm{\Omega }}_1}}} + {H_{{{\rm{\Omega }}_2}}} + {H_s} \hfill\\ 	= {\frac{{{\omega _0}}}{2}{\sigma _z} + {{\rm{\Omega }}_1}{\sigma _x}{\kern 1pt} {\rm{cos}}{\kern 1pt} \left( {{\omega _0}t} \right)} \hfill\\ 	\quad { + {{\rm{\Omega }}_2}{\sigma _y}{\kern 1pt} {\rm{cos}}{\kern 1pt} \left( {{\omega _0}t} \right){\kern 1pt} {\rm{cos}}{\kern 1pt} \left( {{{\rm{\Omega }}_1}t} \right)} + {g{\sigma _x}{\kern 1pt} {\rm{cos}}{\kern 1pt} \left( {{\omega _s}t + \varphi } \right),} \hfill \\ \end{array}$$

where *ω*_0_ is the energy gap of the bare states (*ħ* = 1), *ω*_*s*_ is the frequency of the signal, and *g* is the signal strength which we want to determine. We tune the system, i.e., *ω*_0_, Ω_1_, and Ω_2_, such that *ω*_*s*_ = *ω*_0_ + Ω_1_ + Ω_2_/2.

It is an important feature that phase matching between the signal and the control is not required, which means that the signal phase *φ* can be unknown and moreover, it may vary between experimental runs. In addition, we make the assumption that ${\omega _0} \gg {{\rm{\Omega }}_1} \gg {{\rm{\Omega }}_2} \gg g$. Moving to the interaction picture (IP) with respect to ${H_0} = \frac{{{\omega _0}}}{2}{\sigma _z}$ and making the rotating-wave approximation, we obtain

2 $$\begin{array}{*{20}{l}}\\ {{H_I}} \hfill & = \hfill & {\frac{{{{\rm{\Omega }}_1}}}{2}{\sigma _x} + \frac{{{{\rm{\Omega }}_2}}}{2}{\sigma _y}{\kern 1pt} {\rm{cos}}{\kern 1pt} \left( {{{\rm{\Omega }}_1}t} \right)} \hfill \\ \\ {} \hfill & {} \hfill & { + \frac{g}{2}{\kern 1pt} \left( {{\sigma _ + }{\mathrm{e}^{ - i\left( {\left( {{{\rm{\Omega }}_1} + {{\rm{\Omega }}_2}/2} \right)t + \varphi } \right)}} + {\sigma _ - }{\mathrm{e}^{ + i\left( {\left( {{{\rm{\Omega }}_1} + {{\rm{\Omega }}_2}/2} \right)t + \varphi } \right)}}} \right).} \hfill \\ \end{array}$$

This picture incorporates the effect of Ω_1_ onto a TLS and express the new system in eigenstates of *σ*_*x*_, the $\left| \pm \right\rangle$ (dressed) states, which separates the contributions from Ω_2_ and *g*. For a large enough drive Ω_1_, the $\left| \pm \right\rangle$ eigenstates are decoupled (in first order) from magnetic noise, δ*Bσ*_*z*_, because $\left\langle { \pm \left| {{\sigma _z}} \right| \pm } \right\rangle = 0$. However, power fluctuations δΩ_1_ of Ω_1_ limit the coherence time of the dressed states. The resulting IP is illustrated in the second TLS in Fig. 1c.

We continue by moving to a second IP with respect to ${H_{01}} = \frac{{{{\rm{\Omega }}_1}}}{2}{\sigma _x}$, which leads to

3 $${H_{II}} = \frac{{{{\rm{\Omega }}_2}}}{4}{\sigma _y} + \frac{g}{4}{\kern 1pt} \left( { - i{\sigma _ + }{\mathrm{e}^{ - i\left( {\frac{{{{\rm{\Omega }}_2}}}{2}t + \varphi } \right)}} + i{\sigma _ - }{\mathrm{e}^{ + i\left( {\frac{{{{\rm{\Omega }}_2}}}{2}t + \varphi } \right)}}} \right).$$

Once again, we incorporate Ω_2_ into the dressed states, so that solely the contribution of the signal *g* becomes obvious, which is depicted in the last TLS of Fig. 1c. The second drive, Ω_2_, which is larger than δΩ_1_, creates effectively doubly dressed states (the *σ*_*y*_ eigenstates). These doubly dressed states are immune to power fluctuations of Ω_1_ and hence prolong the coherence time (see Supplementary Note 2 for more details). Moving to the third IP with respect to ${H_{02}} = \frac{{{{\rm{\Omega }}_2}}}{4}{\sigma _y}$ results in

4 $${H_{III}} = \frac{g}{8}{\kern 1pt} \left( {{\sigma _ + }{\mathrm{e}^{ - i\varphi }} + {\sigma _ - }{\mathrm{e}^{ + i\varphi }}} \right),$$

where we can clearly see that the signal *g* induces rotations in the robust qubit subspace (either with *σ*_+_ or *σ*_−_). These rotations are obtained for any value of an arbitrary phase *φ* and the bandwidth (∝1/*T*_2_) is now limited by the coherence time, *T*_2_, of the sensor. Hence, if a given TLS exhibits the possibility of manipulating it via drive fields ${H_{{{\rm{\Omega }}_1}}}$ and ${H_{{{\rm{\Omega }}_2}}}$, we can achieve a high-frequency sensor in the range of *ω*_0_.

By this, we overcome the low frequency limit that is common to state-of-the-art pulsed dynamical decoupling sensing methods. In addition, we present an analog pulsed version of our scheme, where the pulsing rate is much lower than the frequency of the signal (Supplementary Note 9). However it is not a direct measurement of the signal, but based on a signal demodulation approach. Compared to the pulsed schemes, CDD does not suffer from being susceptible to higher harmonics of the decoupling window appearing naturally from the periodic character of the pulsed sequence^46^. Eventually, less power per unit time is used in the continuous scheme leading to a smaller overall noise contribution from the drive.

### Implementation and analysis of the presented scheme

After determining the optimal drive parameters, Ω_1_ and Ω_2_, for the concatenated sensing sequence, and thereby maximize the coherence times, $T_2^{{{\rm{\Omega }}_1}}$ and $T_2^{{{\rm{\Omega }}_1},{{\rm{\Omega }}_2}}$, respectively, of the sensor (Supplementary Note 6), we apply an external high-frequency signal (according to *H*_*s*_ in Eq. (2)) tuned to one of the four appearing energy gaps *ω*_*s*_ of the doubly dressed states. In these energy gaps an effective population transfer can occur between the states of the robust TLS, evidenced by signal induced Rabi oscillations at a rate *g*″ = *g*′/2 = *g*/4 in the double drive case (Supplementary Note 2).

**Fig. 2:**
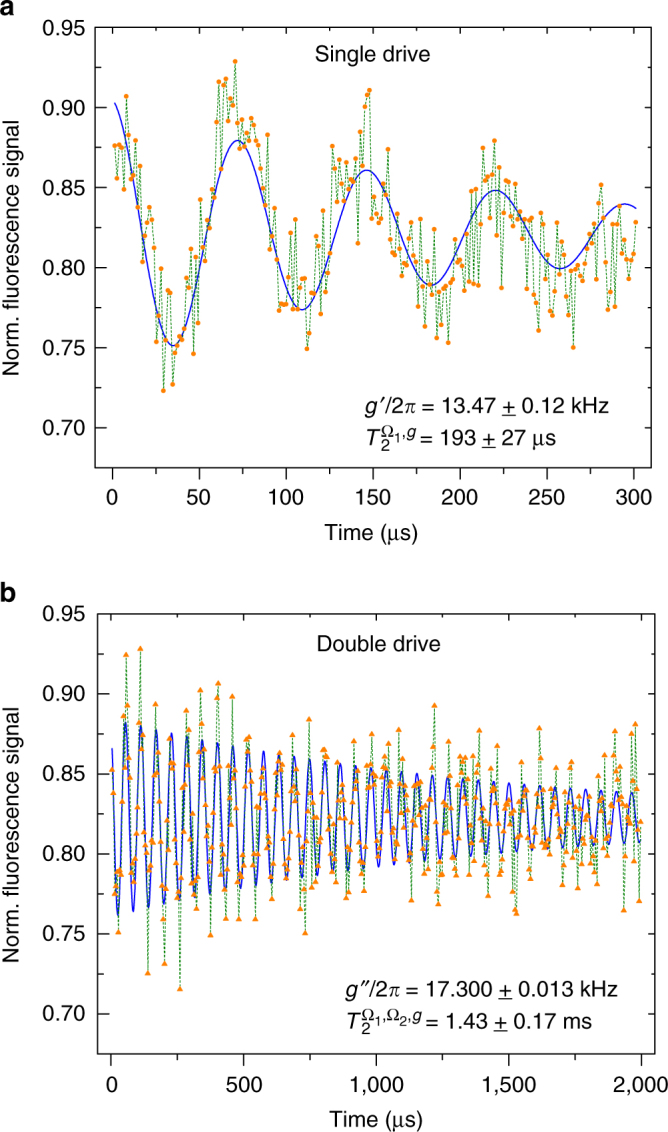
Measurements of an external signal of strength *g*. **a** In a single drive approach with Ω_1_/2*π* = 3.002 MHz a signal *g*′ = *g*/2 is recorded. **b** By the application of two drive fields with Ω_1_/2*π* = 3.363 MHz and Ω_2_/2*π* = 505 kHz, we record a signal *g*″ = *g*/4 and increase the coherence time of the sensor by one order of magnitude with respect to the case of *g* = 0

The measurements take place in the laboratory frame, i.e., all three contributions Ω_1_, Ω_2_, and *g* to the population dynamics of the TLS will be visible. In order to see solely the effect of *g* on the TLS, we alter the modulation of the second drive in Eq. (2) to cos(Ω_1_ *t* + *π*/2). This does not change the performance of the scheme, but only changes the axis of rotation to *σ*_*z*_ for the second drive Ω_2_. Since the readout laser is effectively projecting the population in the *σ*_*z*_ eigenbasis, we can make the Rabi rotations of Ω_2_ invisible to the readout. To remove the effect of Ω_1_ in the data, we can simply sample the measurement at multiple times of ${\tau _{{{\rm{\Omega }}_1}}} = 2\pi {\rm{/}}{{\rm{\Omega }}_1}$, i.e., we measure at times $t = N{\tau _{{{\rm{\Omega }}_1}}}$ ($N \in {\Bbb N}$). This procedure reveals directly *g*″(*g*′) as the signal induces Rabi oscillations of the robust qubit under double (single) drive (Fig. 2). Alternatively, we could have applied at the end of the drive a correction pulse in order to complete the full Ω_1_ and Ω_2_ rotations, so that just the effect of *g*″ remains.

Without a signal, *g*, we achieve coherence times of $T_2^{{{\rm{\Omega }}_1}} \approx 60\,{\rm{\mu s}}$ with a single drive (Ω_2_ = 0, compare Supplementary Fig. 3 in Supplementary Note 6A) and $T_2^{{{\rm{\Omega }}_1},{{\rm{\Omega }}_2}} \approx 393\,{\rm{\mu s}}$ with a double drive (compare Supplementary Fig. 4 in Supplementary Note 6B). The results for long and slow Rabi oscillations induced by an external signal, *g*, under single and double drive (Ω_2_/Ω_1_ ≈ 0.15) are shown in Fig. 2. These illustrate a significant increase of the coherence time of the sensor by two orders of magnitude, from $T_2^{{{\rm{\Omega }}_1}} \approx 60{\kern 1pt} {\rm{\mu s}}$ to a lifetime limited coherence time of (*T*_1_/2≈) $T_2^{{{\rm{\Omega }}_1},{{\rm{\Omega }}_2},g} \approx 1.43\,{\rm{ms}}$. It should be noted that the signal itself can be considered as an additional drive (cf. Eq. (3)), correcting external errors δΩ of the previous drive and thereby prolonging the coherence time even further. Consequently, we can improve the bandwidth for high-frequency sensing by almost three orders of magnitude from ~900 kHz (for $T_2^* \approx 1.1\,{\rm{\mu s}}$) to ~700 Hz (for a $T_2^{{{\rm{\Omega }}_1},{{\rm{\Omega }}_2},g} \approx 1.43\,{\rm{ms}}$). Moreover, in Supplementary Note 3 we discuss an improved version of our scheme which has the potential to push the coherence time of the sensor further towards the lifetime limit.

To benchmark the double drive scheme against a standard single drive approach, we determine the smallest magnetic field which can be sensed after an accumulation time *t*. The smallest measurable signal *S* is eventually bounded by the smallest measurable magnetic field change δ*B*_min_, which is found to be

5 $${\rm{\delta }}{B_{{\rm{min}}}}(t,\tau ) = \frac{{{\rm{\delta }}S}}{{{\rm{max}}{\kern 1pt} \left| {\frac{{\partial S}}{{\partial B}}} \right|}} = \frac{1}{{{\gamma _{{\rm{NV}}}}}}\frac{{\sigma (t)}}{{\alpha \tau C}}.$$

Here, *γ*_NV_/2*π* = 28.8 GHz T^−1^ is the gyromagnetic ratio of the NV defect, *σ*(*t*) is the standard deviation of the measured normalized fluorescence counts after time *t*, *α* accounts for a different phase accumulation rate depending on the decoupling scheme, and *C* is the contrast of the signal (see Supplementary Note 5 for detailed derivation). Since the photon counting is shot noise limited, we have $\sigma (t) = 1{\rm{/}}\sqrt {{N_{{\rm{ph}}}} \cdot N}$, with *N*_ph_ being the number of photons measured in *τ* and *N* = *t*/*τ* is the number of sequence repetitions. With this, Eq. (5) will transform in the commonly known form^47, 48^ with some measurement dependent constants.

**Fig. 3:**
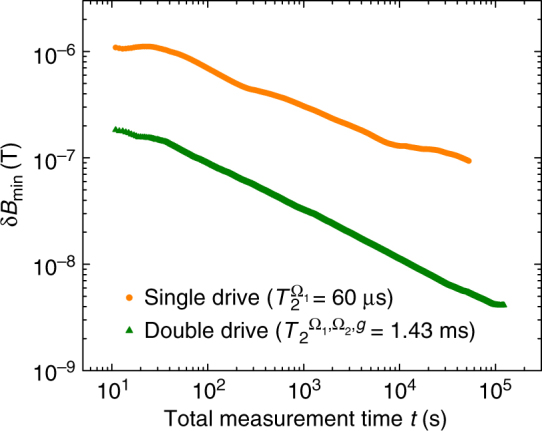
Comparison of the smallest measurable magnetic field change δ*B*_min_ = (2*π*δ*g*_min_)/*γ*_NV_ as a function of total measurement time. To show the total improvement, we obtain *σ*(*t*) at $\tau = T_2^{{{\rm{\Omega }}_1}} \approx 60\,{\rm{\mu s}}$ in the single drive case and *σ*(*t*) at $\tau = T_2^{{{\rm{\Omega }}_1},{{\rm{\Omega }}_2},g} \approx 1.43\,{\rm{ms}}$ in the double drive case. Note, that for both data traces a signal was always present, *g*/2*π* = 26.9 kHz and *g*/2*π* = 69.2 kHz in the single and in the double drive, respectively. But only in the double drive the coherence time prolonging effect of *g* was included into the choice of *τ* for Eq. (5) (i.e., the measurement was performed at $\tau = T_2^{{{\rm{\Omega }}_1},{{\rm{\Omega }}_2},g}$ instead at $\tau = T_2^{{{\rm{\Omega }}_1},{{\rm{\Omega }}_2}} \approx 393\,{\rm{\mu s}}$)

We recorded *σ*(*t*) as a function of time and use this to determine δ*B*_min_. The results of this measurement for both the single and double drive are summarized in Fig. 3. The sensitivity can be obtained by $\eta (\tau ) = {\rm{\delta }}{B_{{\rm{min}}}}(t,\tau )\sqrt t$, which is optimal in the vicinity of the coherence time of the sensor, *τ* ≈ *T*_2_. With our system, we achieve a sensitivity of ${\eta _{{{\rm{\Omega }}_1},{{\rm{\Omega }}_2},g}}$ ≲1 μT Hz^−0.5^ in the double drive case at ~1.6 GHz, which should be compared to ${\eta _{{{\rm{\Omega }}_1},g}}$ ≲20 μT Hz^−0.5^ for a single drive approach.

Both traces in Fig. 3 were recorded while a signal *g* was applied. Apart from the mere fact, that the number of driving fields are different, the specific choice for *τ* will also determine the magnitude of the smallest measurable magnetic field change δ*B*_min_. Obtaining the coherence time without a signal, *g*, (which are $T_2^{{{\rm{\Omega }}_1}}$ and $T_2^{{{\rm{\Omega }}_1},{{\rm{\Omega }}_2}}$), is a common practice in the field, but will not result in a correct choice of *τ* for the sensitivity measurement and also for Eq. (5), since the signal has an impact on the sensor’s sensitivity. However, if *δB*_min_ shall be evaluated correctly, then the non-linearity, i.e., the coherence time prolonging effect of the signal, has to be taken into account. Otherwise an even worse δ*B*_min_ will be measured as it is exemplarily shown for the single drive case in Fig. 3, where δ*B*_min_ was evaluated and measured under the naive assumption that the signal has no effect on the coherence time of the sensor (i.e., we measure at $\tau = T_2^{{{\rm{\Omega }}_1}}$ and not at $\tau = T_2^{{{\rm{\Omega }}_1},g}$). This effect was included in the double drive case.

**Fig. 4:**
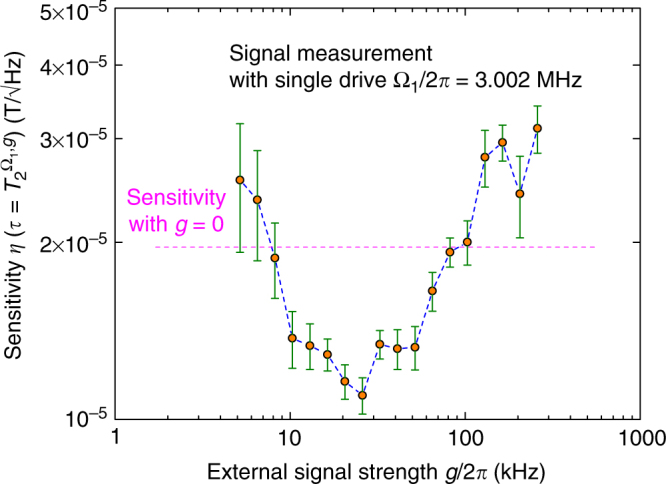
Projected sensitivity of the single drive scheme as a function of signal strength, *g*. The coherence time measurements, $T_2^{{{\rm{\Omega }}_1},g}$ for a fixed drive Ω_1_ and subsequently increasing signal strength, *g*, (displayed in Supplementary Fig. 5) are expressed in terms of sensitivity. The magenta dashed line indicates the sensitivity of the sensor if no signal is applied. The figure illustrates that an external signal has a non-linear effect on the sensitivity of the sensor, which has to be taken into account in the sensitivity estimation. The error bars ${\rm{\Delta }}\eta \left( {T_2^{{{\rm{\Omega }}_1},g}} \right)$ in this graph represent the converted standard deviation ${\rm{\Delta }}T_2^{{{\rm{\Omega }}_1},g}$ from Supplementary Fig. 5

To examine the signal protection effect more in detail, the coherence time of the sensor is measured as a function of signal strength, *g*, in a single drive configuration (Supplementary Note 7). From these measurements we project the sensitivity associated with a specific signal strength (Fig. 4), assuming *σ*(*t*) is unchanged for the same repetition *N*. This is a reasonable assumption given that the only difference between measurements is the signal strength, *g*, and sequence length, *τ*.

Eventually, this phenomenon, which seems to be an inherent part of this continuous scheme, can be used to further increase the performance of the sensor by fine tuning the controlled parameters (static bias field *B*_bias_ and thereby changing *ω*_0_, Ω_1_ and Ω_2_) to match the signal frequency, *ω*_*s*_, and strength, *g* (see Supplementary Note 3 for further discussions).

## Discussion

We have demonstrated that dynamical decoupling can be used in the context of sensing high frequency fields. In contrast to state-of-the-art pulsed dynamical decoupling protocols, we can show that CDD can be simultaneously integrated into the sensing task. By utilizing a NV center in diamond we have demonstrated by pure concatenation of two drives a coherence time of ~393 μs which constitutes an improvement of more than two orders of magnitude over $T_2^*$, and an increase of resolution from the MHz to a few kHz. The application of this method for wireless communication^49^ could have a transformative effect due to the high resolution of the protocol. Since the protocol is applicable to a variety of solid-state, molecular, and atomic systems, we believe that it has a great potential to have a significant impact on many fields and tasks that involve high frequency sensing (up to frequencies in the THz range). Eventually, this method could also be used to improve the coupling to quantum systems^30^. We would like to note that during the preparation of this manuscript we became aware of a related independent work by Joas et al.^50^.

### Data availability

The authors declare that all relevant data supporting the findings of this study are available within the paper (and its Supplementary Information file). Any raw data can be obtained from the corresponding authors on reasonable request.

## Electronic supplementary material

Supplementary Information

Peer Review File
