# Supplementary material for: Narrow-bandwidth sensing of high-frequency fields with continuous dynamical decoupling
Source: Nat Commun. 2017 Oct 19;8:1105. doi: 10.1038/s41467-017-01159-2 (PMC5648921; doi:10.1038/s41467-017-01159-2)
Supplement: Supplementary file 1 — Supplementary Information [file 41467_2017_1159_MOESM1_ESM.pdf]

Supplementary Note 1: Sensing of a high frequency signal with a single drive

In this section we describe in detail how the enhanced sensing of high frequency signals can be performed with a two-level system (TLS) by employing a single continuous driving field. The Hamiltonian of the TLS is given by

$$H=\frac{\omega_0}{2}\sigma_z+\Omega_1\left(1+\delta_{\Omega_1}(t)\right)\cos(\omega_0 t)\sigma_x+g\cos(\omega_s t+\varphi)\sigma_x+\delta_B(t)\sigma_z, \tag{1}$$

where ω_0 corresponds to the energy gap of the bare states, Ω_1 is the Rabi frequency of the on-resonance driving field, g is the Rabi frequency of the signal, ω_s is the frequency of the signal, and φ is a random phase, which indicates that the signal field and the driving field are not phased-matched, and therefore φ has a different value in each experiment. We tune the system, i.e., ω_0 and Ω_1, such that $\omega_s=\omega_0+\Omega_1$. In addition, $\delta_{\Omega_1}(t)\Omega_1$ represents the fluctuations of the driving field, and $\delta_B(t)$ is the magnetic noise. Moving to the interaction picture (IP) with respect to $H_0=\frac{\omega_0}{2}\sigma_z$ and, assuming that $\omega_0\gg\Omega_1$, making the rotating-wave-approximation (RWA) we get

$$H_I=\frac{\Omega_1}{2}\left(1+\delta_{\Omega_1}(t)\right)\sigma_x+\frac{g}{2}\left(\sigma_+e^{-i(\Omega_1 t+\varphi)}+\sigma_-e^{+i(\Omega_1 t+\varphi)}\right)+\delta_B(t)\sigma_z. \tag{2}$$

In the basis of the dressed states, the σ_x eigenstates, the Hamiltonian reads ($x\to z$, $z\to -x$, $y\to y$)

$$H_I=\frac{\Omega_1}{2}\left(1+\delta_{\Omega_1}(t)\right)\sigma_z+\frac{g}{2}\left(\cos(\Omega_1 t+\varphi)\sigma_z+\sin(\Omega_1 t+\varphi)\sigma_y\right)-\delta_B(t)\sigma_x. \tag{3}$$

Because the magnetic noise couples between the dressed states, in first order, the noise induces a longitudinal relaxation (decay) rate of $\sim S_{BB}(\Omega_1)$, where S_{BB} is the power spectrum of the noise. A large enough Ω_1 ensures that the longitudinal relaxation rate is negligible ($S_{BB}(\Omega_1)\ll 1/T_1$). In this case, within the experiment time the noise does not induce transitions between the dressed states, but does result in a second order fluctuating phase shift of $\sim\delta_B^2(t)/\Omega_1$. The resulting dephasing rate is considerably diminished with an increasing Ω_1 [1]. The main limitation on the coherence time of the dressed states, $T_2^{\Omega_1}$, is due to power fluctuations of the driving field, $\delta_{\Omega_1}(t)\Omega_1$, which limit the coherence time to $T_2^{\Omega_1}\sim 1/(\delta_{\Omega_1}(t)\Omega_1)$. For typical experimental setups $\delta_{\Omega_1}(t)\sim 0.1-1\%$ implies an improvement of ~ 1 order of magnitude in the coherence time compared to T_2^*.

Note, that throughout this manuscript and in the main text, we use the following notation: T_1, known as the longitudinal relaxation time of the qubit, is called the lifetime of the sensor. T_2^* describes the pure dephasing time of the sensor, if no protection or drive is applied to the sensor. T_2 denotes the transverse relaxation time, which is the coherence time in a pulsed dynamical decoupling experiments. $T_2^{\Omega_1}$ expresses the coherence time under drive Ω_1 and $T_2^{\Omega_1,g}$ characterize a coherence time under drive Ω_1 with an externally applied signal g.

We continue by moving to the interaction picture (IP) with respect to $H_{01}=\frac{\Omega_1}{2}\sigma_z$ (in the basis of the dressed states) and, assuming that $\Omega_1\gg g$, making the rotating-wave-approximation (RWA), which leads to

$$H_{II}=\frac{g}{4}\left(-i\sigma_+e^{-i\varphi}+i\sigma_-e^{+i\varphi}\right)+\frac{\delta_{\Omega_1}(t)\Omega_1}{2}\sigma_z, \tag{4}$$

where we neglected the (fast rotating) terms of the magnetic noise. The signal in H_{II} corresponds to the on-resonance coupling between the dressed states, and hence the signal induces rotations of the dressed qubit, regardless to the value of φ (so long as φ is constant during a single experiment).

Supplementary Note 2: Sensing of a high frequency signal with a double drive

In order to mitigate the driving fluctuations of the (first) driving field we introduce a second drive, polarized along a perpendicular direction with respect to the polarization of the first driving field. We therefore consider the Hamiltonian

$$H=\frac{\omega_0}{2}\sigma_z+\Omega_1\left(1+\delta_{\Omega_1}(t)\right)\cos(\omega_0 t)\sigma_x+\Omega_2\left(1+\delta_{\Omega_2}(t)\right)\cos(\omega_0 t)\cos(\Omega_1 t)\sigma_y+g\cos(\omega_s t+\varphi)\sigma_x+\delta_B(t)\sigma_z, \tag{5}$$

where Ω_2 is the Rabi frequency of the second drive, and we tune the system such that $\omega_s = \omega_0 + \Omega_1 + \frac{\Omega_2}{2}$. In addition, we assume that $\omega_0 \gg \Omega_1 \gg \Omega_2 \gg g$. In the first IP with respect to $H_0 = \frac{\omega_0}{2}\sigma_z$, and after making the RWA and moving to the basis of the dressed states ($x \to z$, $z \to -x$, $y \to y$) we have that

$$H_I = \frac{\Omega_1}{2}\left(1+\delta_{\Omega_1}(t)\right)\sigma_z + \frac{\Omega_2}{2}\left(1+\delta_{\Omega_2}(t)\right)\cos(\Omega_1 t)\,\sigma_y + \frac{g}{2}\left(\cos\left(\left(\Omega_1+\frac{\Omega_2}{2}\right)t+\varphi\right)\sigma_z + \sin\left(\left(\Omega_1+\frac{\Omega_2}{2}\right)t+\varphi\right)\sigma_y\right) - \delta_B(t)\,\sigma_x. \tag{6}$$

We continue by moving to the second IP with respect to $H_{01} = \frac{\Omega_1}{2}\sigma_z$ (in the basis of the dressed states) and taking the RWA to obtain

$$H_{II} = \frac{\delta_{\Omega_1}(t)\,\Omega_1}{2}\sigma_z + \frac{\Omega_2}{4}\left(1+\delta_{\Omega_2}(t)\right)\sigma_y + \frac{g}{4}\left(\sigma_+ e^{-i\left(\frac{\Omega_2}{2}t+\varphi\right)} + \sigma_- e^{+i\left(\frac{\Omega_2}{2}t+\varphi\right)}\right), \tag{7}$$

where we neglected the (fast rotating) terms of the magnetic noise. In the basis of the doubly-dressed states, the σ_y eigenstates ($y \to z$, $z \to -y$, $x \to x$), the Hamiltonian is given by

$$H_{II} = \frac{\Omega_2}{4}\left(1+\delta_{\Omega_2}(t)\right)\sigma_z - \frac{\delta_{\Omega_1}(t)\,\Omega_1}{2}\sigma_y + \frac{g}{4}\left(\cos\left(\frac{\Omega_2}{2}t+\varphi\right)\sigma_x + \sin\left(\frac{\Omega_2}{2}t+\varphi\right)\sigma_z\right). \tag{8}$$

Finally, in the third IP with respect to $H_{02} = \frac{\Omega_2}{4}\sigma_z$ (in the basis of the doubly-dressed states), and after making the RWA we have that

$$H_{III} = \frac{g}{8}\left(\sigma_+ e^{-i\varphi} + \sigma_- e^{+i\varphi}\right) + \frac{\delta_{\Omega_2}(t)\,\Omega_2}{4}\sigma_z, \tag{9}$$

where we neglected the (fast rotating) terms of the driving fluctuations of Ω_1, which now contribute only as a second order effect. We therefore conclude that the signal in H_{III} corresponds to the on-resonance coupling between the doubly-dressed states, and hence the signal induces rotations of the doubly-dressed qubit. The doubly-dressed stated are vulnerable to fluctuations of Ω_2, but since $\Omega_2 \ll \Omega_1$ these fluctuations have a smaller effect than fluctuations in Ω_1; with $\delta_{\Omega_i}(t) \sim 0.1 - 1\%$ the coherence time is improved by ~ 1 order of magnitude with respect to the coherence time of the dressed states (an improvement of ~ 2 orders of magnitude with respect to T_2^*). In principle, the robustness to driving fluctuations can be further improved by the concatenation of more driving fields [2].

Supplementary Note 3: Improved scheme

In our scheme, a weak signal may further prolong the coherence time of the probe qubit. To see this we continue from equation (9) and assume for simplicity that $\varphi = 0$. In this case

$$H_{III} = \frac{g}{8}\sigma_x + \frac{\delta_{\Omega_2}(t)\,\Omega_2}{4}\sigma_z. \tag{10}$$

However, the difference between the signal, $g\cos(\omega_s t + \varphi)\,\sigma_x$, and a concatenated third drive, which in our case could be given by $\Omega_3 \cos(\omega_0 t)\cos((\Omega_2/2)\,t)\,\sigma_x$, comes from the counter-rotating terms that we usually neglect when making the RWA. Although that the condition $\Omega_1 \gg \Omega_2 \gg g$ holds, examination of the counter-rotating terms of the signal g, the magnetic noise $\delta_B(t)$, and the driving noise of the first drive $\delta_{\Omega_1}(t)$, in the third IP, reveals that there are counter-rotating terms of g and $\delta_B(t)$ and of g and $\delta_{\Omega_1}(t)$ that have identical frequencies. This implies that these terms result in an effective time-independent Hamiltonian [3] that should be included in equation (10), and hence,

$$H_{III} = \frac{g}{8}\sigma_x + \frac{\delta_{\Omega_2}(t)\,\Omega_2}{4}\sigma_z + \frac{g\,\delta_B(t)}{8\Omega_1}\sigma_x + \frac{g\,\delta_{\Omega_1}(t)}{4\Omega_2}\sigma_x. \tag{11}$$

The added effective terms imply dephasing rates of $\Gamma_{\delta_B(t)} = \frac{g}{8\Omega_1}S_{BB}(0)$ and $\Gamma_{\delta_{\Omega_1}(t)} = \frac{g}{4\Omega_2}S_{\Omega_1\Omega_1}(0)$, where $S_{BB}(\omega)$ and $S_{\Omega_1\Omega_1}(\omega)$ are the power spectra of the magnetic noise and the noise of the first driving field, respectively. Hence, increasing values of g result in increasing dephasing rates. This was verified in simulations (see Supplementary Note 8), and to

some extent experimentally as shown in Supplementary Fig. 5 and in Fig. 4 of main text. Indeed, for small enough values of g this second-order effect is negligible and the signal prolongs the coherence time. For large values of g the coherence time is decreased, even below the coherence time of the doubly-dressed states.

In order to circumvent this problem, we tune the system such that $\omega_s = \omega_0 + \frac{\Omega_2}{2}$ instead of $\omega_s = \omega_0 + \Omega_1 + \frac{\Omega_2}{2}$, which means that we now utilize a different transition in order to couple between the doubly-dressed states (see Supplementary Fig. 1b). In this case, in the first IP with respect to $H_0 = \frac{\omega_0}{2}\sigma_z$, and after making the RWA and moving to the basis of the dressed states we have that

$$H_I = \frac{\Omega_1}{2}\left(1+\delta_{\Omega_1}(t)\right)\sigma_z + \frac{\Omega_2}{2}\left(1+\delta_{\Omega_2}(t)\right)\cos\left(\Omega_1 t\right)\sigma_y + \frac{g}{2}\left(\cos\left(\frac{\Omega_2}{2}t+\varphi\right)\sigma_z + \sin\left(\frac{\Omega_2}{2}t+\varphi\right)\sigma_y\right) - \delta_B(t)\,\sigma_x. \tag{12}$$

Moving now to the second IP with respect to $H_{01} = \frac{\Omega_1}{2}\sigma_z$ (in the basis of the dressed states) and taking the RWA we obtain

$$H_{II} = \frac{\delta_{\Omega_1}(t)\,\Omega_1}{2}\sigma_z + \frac{\Omega_2}{4}\left(1+\delta_{\Omega_2}(t)\right)\sigma_y + \frac{g}{2}\cos\left(\frac{\Omega_2}{2}t+\varphi\right)\sigma_z, \tag{13}$$

where we neglected the (fast rotating) terms of the magnetic noise. In the basis of the doubly-dressed states, the σ_y eigenstates ($y \to z$, $z \to -y$, $x \to x$), the Hamiltonian is given by

$$H_{II} = \frac{\Omega_2}{4}\left(1+\delta_{\Omega_2}(t)\right)\sigma_z - \frac{\delta_{\Omega_1}(t)\,\Omega_1}{2}\sigma_y - \frac{g}{2}\cos\left(\frac{\Omega_2}{2}t+\varphi\right)\sigma_y. \tag{14}$$

Finally, in the third IP with respect to $H_{02} = \frac{\Omega_2}{4}\sigma_z$ (in the basis of the doubly-dressed states), and after making the RWA we have that

$$H_{III} = \frac{g}{4}\left(i\sigma_+ e^{-i\varphi} - i\sigma_- e^{+i\varphi}\right) + \frac{\delta_{\Omega_2}(t)\,\Omega_2}{4}\sigma_z, \tag{15}$$

Here, the calculation of the effective Hamiltonian of the counter-rotating terms yields (taking $\varphi = 0$ again)

$$H_{III} = -\frac{g}{4}\sigma_y + \frac{\delta_{\Omega_2}(t)\,\Omega_2}{4}\sigma_z + \frac{g\delta_B(t)}{8\Omega_1}\sigma_z. \tag{16}$$

In this case the second order contribution of the driving noise, $g\delta_{\Omega_1}(t)$, vanishes, and because the second order contribution of the magnetic noise, $g\delta_B(t)$, is perpendicular to the signal, it contributes in higher orders only (assuming $\Omega_1 \gg |\delta_B(t)|$). Therefore, the improved scheme results in even prolonged coherence times and enables the measurement of stronger signals. Moreover, the measured signal in this scheme is stronger by a factor of 2 compared to the original scheme. A comparison between the schemes is shown in Supplementary Note 8 where the results of the simulations suggest that the improved scheme may increase the coherence time by an additional order of magnitude.

Supplementary Note 4: Detailed level scheme and setup

The diamond used for this measurements contains natural abundance of ^{13}C (1.1%) and the selected nitrogen-vacancy (NV) was situated roughly 2 µm below the surface. All the measurements are performed at a static magnetic field of $B_{\text{bias}} = 446\,$G, where the Nitrogen nuclear spin of the NV is polarized [4], so that the hyperfine transitions appearing from the coupling to the ^{14}N do not intervene with the protocols. The static magnetic field is aligned parallel to the quantization axis of the NV centre (connecting the nitrogen and the vacancy) and is defined as the z-axis of the system. Supplementary Fig. 1a shows an optically detected magnetic resonance (ODMR) measurement from where ω_0 is determined. A more detailed level scheme

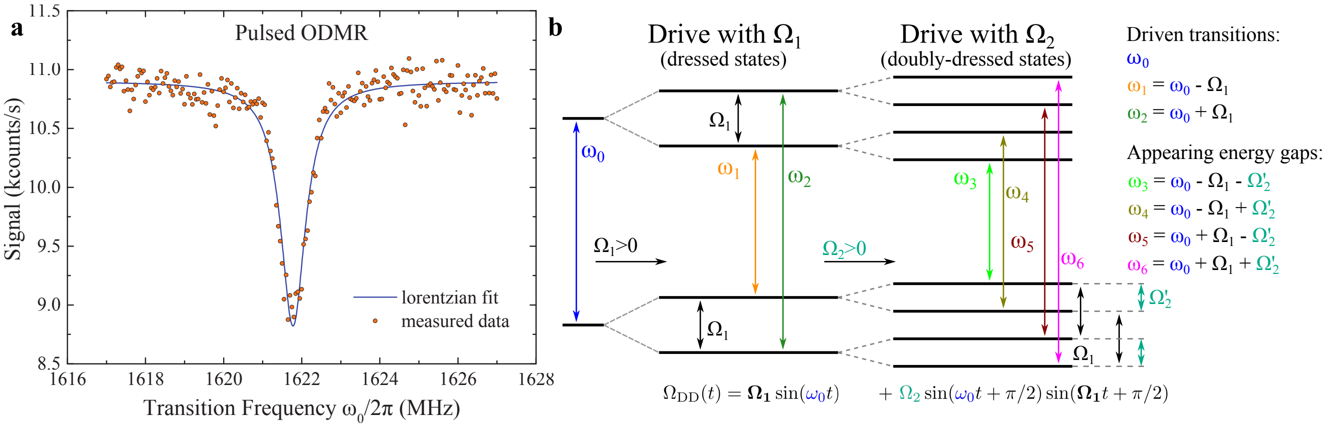

Supplementary Figure 1. **Characterization of the NV system by probing the TLS and a detailed level scheme of the double drive.** (a) A pulsed ODMR measurement, where the pulse length of the applied microwave corresponds to $\tau_{\Omega_1}/2 \approx 1.08\,\mu$s and the frequency was swept. If the right energy gab ω_0 between the TLS is hit by the microwave, an effective transfer of population from the bright to the dark state can occur which becomes visible in a frequency dependent decrease in fluorescence counts. (b) A detailed level scheme of the double drive procedure with the appearing energy gabs and the driven transitions. Note that in the modulation of the second drive, Ω_2, a $\pi/2$ was inserted to make Ω_2 not visible in the readout (cf. equation (1) in the main text).

containing the effect of two applied drives to the TLS of the NV centre is depicted in Supplementary Fig. 1b.

By switching on the first drive Ω_1 a dressed state configuration is obtained, where the new eigenstates are separated by Ω_1. In the single drive configuration the energy levels ω_1 and ω_2 are susceptible to external signal. The dressed states are decoupled from external magnetic noise δB, but suffer mainly from the drive noise $\delta\Omega_1$. To decouple the sensor from $\delta\Omega_1$ noise, a second drive Ω_2 of the order of $\delta\Omega_1$ is applied, which drives effectively the appearing transitions ω_1 and ω_2 due to

$$\sin\left(\omega_0 + \frac{\pi}{2}\right)\sin\left(\Omega_1 + \frac{\pi}{2}\right) = \frac{1}{2}\Big(\cos\left(\omega_0 - \Omega_1\right) + \cos\left(\omega_0 + \Omega_1\right)\Big) \tag{17}$$

leading to a doubly-dressed state configuration. Here, four new energy gaps $\omega_s = \omega_0 \pm \omega_1 \pm \omega_2'$ are opened, which can be addressed by an external signal. Note that due to a change in interaction pictures $\omega_2' = \omega_2/2$. Consequently, the doubly-dressed state suffers mainly from noise contributions of $\delta\Omega_2$.

This concatenation of drives can be continued and will increase the coherence time of the sensor until the noise $\delta\Omega_i$ of the additional applied i-th drive field is on the order of the drive Ω_i itself, or until T_1 time of the bare states of the sensor is reached.

Both drives, Ω_1 and Ω_2, are sampled and outputted from one channel of an AWG (arbitrary waveform generator, Keysight M8195A) with a time resolution of 65 GS/s. The DAC (digital analog converter) in this device has a resolution of 8bit per channel at maximum 1V (peak-to-peak). As an external signal source, the Rohde&Schwarz SMIQ03B was used.

Supplementary Note 5: Calculating the slope

The sensor obtains a phase ϕ during a time t which can be written as

$$\phi(t) = \int_0^t \gamma_{\mathrm{NV}} \alpha B \mathrm{d}\tau = \gamma_{\mathrm{NV}} \alpha B t \tag{18}$$

if B remains constant over time. α is a constant factor, which depends on the measurement scheme (and determines the rate of phase accumulation). For a measurement of B under single drive we can set $\alpha_{\mathrm{sd}} = 1/2$ and for the double drive case $\alpha_{\mathrm{dd}} = 1/4$. This can be seen in equation (9), where the rate at which the signal is recorded is reduced in the double drive case to $(g/2) \cdot \alpha_{\mathrm{dd}} = g/8$.

In the measurement a normalized fluorescence signal S is recorded, where the state $\phi = 0$ is associated with the bright state of the NV (which corresponds to a normalized fluorescence value b). The state $\phi = \pi$ is the dark state of the NV (which corresponds to a normalized fluorescence value d). Therefore the signal accumulation corresponds to

$$S = \frac{(b+d)}{2} + \frac{(b-d)}{2} \cos(2\phi)\,. \tag{19}$$

since for a TLS we have $S \propto \cos^2(\phi)$.

An unknown magnetic field amplitude B can be extracted by performing a measurement of S. The uncertainty δS of the signal S will eventually determine the error in δB, which are connected by

$$\delta S = \frac{\partial S}{\partial B} \delta B\,. \tag{20}$$

The sensor is the most sensitive to a small change in the magnetic field at the point where the signal S has the maximum change, which is the maximal slope

$$\begin{aligned} \max \left| \frac{\partial S}{\partial B} \right| &= \max \left| -\frac{(b-d)}{2} \sin(2\gamma_{\mathrm{NV}} \alpha B \tau) \cdot 2\gamma_{\mathrm{NV}} \alpha \tau \right| \\ &= (b-d)\gamma_{\mathrm{NV}} \alpha \tau = \omega_S C \end{aligned} \tag{21}$$

Here the full amplitude $C = (b-d)$ of the signal and the rate of change $\omega_S = \gamma_{\mathrm{NV}} \alpha \tau$ in S were introduced. Since S represents a normalized value (normalized with the bright state of the sensor) C directly corresponds to the contrast of the signal. Note that the time (for one measurement run) τ will determine the magnitude of the slope.

To obtain the state of the NV we will count photons. Since the number of photons in one measurement run τ is very small for a NV, we will have to repeat the measurement $N = t/\tau$ times. Each photon record becomes an independent measurement and the uncertainty of the state signal δS is Poissonian distributed, $\delta S(t) = 1/\sqrt{N_{\mathrm{ph}} \cdot N} = \sigma(t)$. N_{ph} represent the amount of photons counted in one experimental run τ, which can be understood as $N_{\mathrm{ph}} = \Gamma_c \tau$, with Γ_c being the count rate.

In the measurement we will accumulate a signal S for a time t and determine the standard deviation $\sigma(t)$ of the accumulated signal over time. Therefore the minimum resolvable magnetic field writes

$$\delta B_{\mathrm{min}}(t,\tau) = \frac{\delta S}{\max \left| \frac{\partial S}{\partial B} \right|} = \frac{1}{\gamma_{\mathrm{NV}}} \frac{\sigma(t)}{\alpha \tau C} = \frac{1}{\gamma_{\mathrm{NV}}} \frac{1}{\alpha C} \frac{1}{\sqrt{N_{\mathrm{ph}} \tau t}} \tag{22}$$

The sensitivity η for a repetitive measurement at τ after time t (i.e. after repeating the measurement $N = t/\tau$ times) is consequently

$$\eta(\tau) = \delta B_{\mathrm{min}}(t,\tau)\sqrt{t} = \frac{1}{\gamma_{\mathrm{NV}}} \frac{1}{\alpha C} \frac{1}{\sqrt{N_{\mathrm{ph}} \tau}} \tag{23}$$

As the signal of the sensor decays with t resulting in a reduced contrast C, the optimal measurement point τ will be situated at the coherence time T_2, where also the contrast C of the signal should be determined and we will end up in

$$\eta(T_2) = \frac{\hbar}{g\mu_B} \frac{1}{\alpha C} \frac{1}{\sqrt{N_{\mathrm{ph}} T_2}} \tag{24}$$

where $\gamma_{\rm NV} = g\mu_B/\hbar$ was used. A similar derivation can be found in [5]. Note that in general the contrast $C = C(T_2)$ of the signal is depending on the coherence time of the sensor, which mean that it will decay for a time $t > T_2$, which will cause a worst sensitivity η. The appearing constants $1/(\alpha C)$ are measurement related values.

Supplementary Fig. 2a shows a measurement to determine the maximal slope $|\partial S/\partial B|$ in the double drive case, where an externally applied signal was varied in strength g. With this result it is possible to obtain via $\delta B_{\rm min} = (2\pi\delta g_{\rm min})/\gamma_{\rm NV}$ directly $\delta B_{\rm min}(t,\tau)$ by measuring at the point of the maximal slope $\sigma(t)$ over time t. The result is plotted in Fig. 3 of the main text.

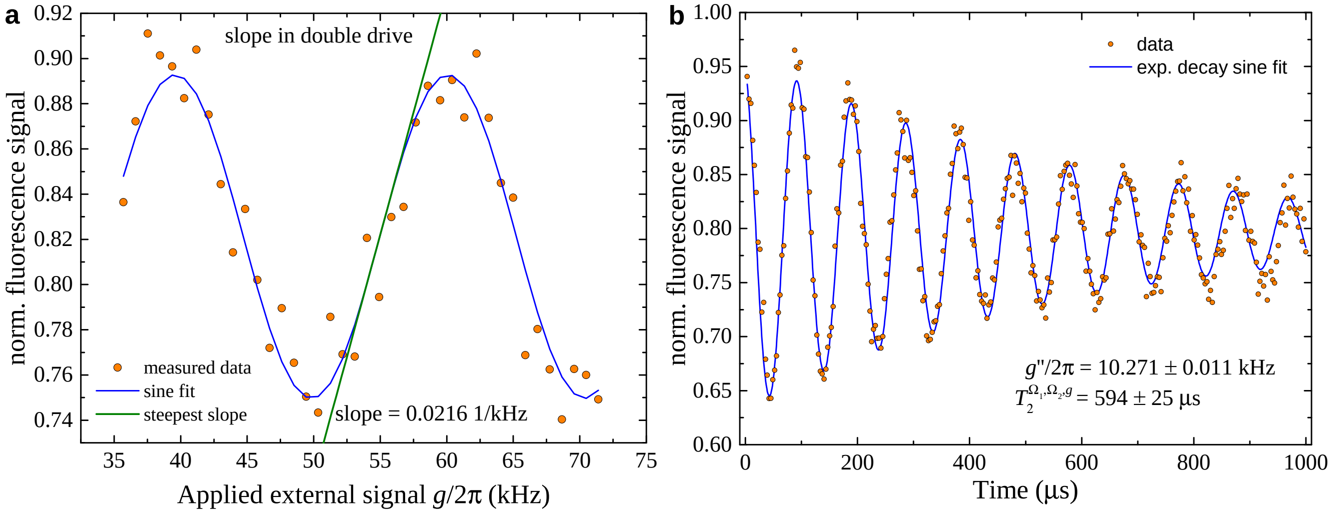

Supplementary Figure 2. **Two different approaches to determine $\delta B_{\rm min}$**. (a) Direct slope measurement in double drive at $\tau = 250\,\mu$s by varying the applied signal strength g. From this measurement the maximal slope $|\partial S/\partial B|_{\rm max}$ can be obtained. (b) Coherence time measurement in double drive with a signal g. A signal of strength $g/2\pi \approx 41$ kHz was applied during a double drive measurement with $\Omega_1/2\pi = 3.366$ MHz and $\Omega_2/2\pi = 519.5$ kHz. In the double drive $g'' = g/4$ is measured. The total measurement took about 1 day (where a signal-to-noise ratio of ≈ 66 was obtained).

Alternatively, it is possible to obtain $\delta B_{\rm min}(t,\tau)$ by measuring the coherence time under drive with a signal, $\tau = T_2^{\Omega_1,\Omega_2,g}$, and the resulting contrast, C, at the time $T_2^{\Omega_1,\Omega_2,g}$ like in Supplementary Fig. 2b, to obtain the denominator of equation (22).

Supplementary Note 6: Determine the optimal drive parameters

A. Optimal drive parameter in single drive

Before an external high frequency signal can be measured suitable values for the drive fields Ω_1 and Ω_2 have to be chosen, to obtain the maximal coherence time of the sensor. Since the first drive Ω_1 increases the decoupling of the sensor from the environment but introduces drive noise $\delta\Omega_1$ with a stronger drive Ω_1, an optimal value has to be selected. The optimal value should prolong the coherence time of the sensor as much as possible for a given drive configuration. Supplementary Fig. 3 shows Rabi (= single drive) measurements with different drive strength, Ω_1, displayed against the extracted coherence time of the sensor under drive, $T_2^{\Omega_1}$. It becomes obvious that for a slower drive (< 1 MHz) the coherence time of the sensor, $T_2^{\Omega_1}$,

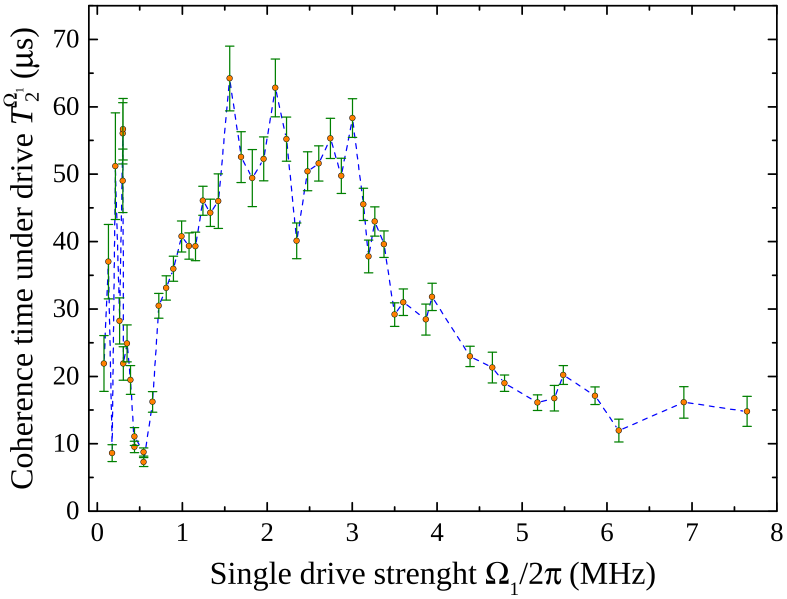

Supplementary Figure 3. **Measurement of the coherence time, $T_2^{\Omega_1}$, of the qubit under an increasing drive,** Ω_1. For every displayed value of the drive, Ω_1, a Rabi measurement was performed until it was decayed. The resulting curve was fitted to $f(t) = c + \alpha \sin(\Omega_1 t + \phi) \exp(t/T_2^{\Omega_1})$ to extract the coherence time $T_2^{\Omega_1}$. The error bars represent the standard deviation $\Delta T_2^{\Omega_1}$ of the coherence time in the fit.

is not increasing monotonically. One of the reason for this is a less efficient decoupling by Ω_1 allowing various interactions between individual nearby ^{13}C nuclear spins and the overall ^{13}C bath contribution. For a static magnetic field of $B = 446$ G and $\gamma_{^{13}C}/2\pi = 1.0705$ kHz/G we expect the Larmor frequencies of the ^{13}C bath to be around $\nu_{\mathrm{larmor}^{13}\mathrm{C}} = \gamma_{^{13}C} B \approx 477$ kHz, which fits well with the large dip at around 500 kHz. To use the dressed states as a high frequency sensor under a single drive, we select $\Omega_1/2\pi > 2.5$ MHz based on Supplementary Fig. 3 to make sure that a sufficient decoupling from the environment can be guaranteed. Compared to the pure dephasing time of this NV with $T_2^* = 1.1$ μs, the single drive with $\Omega_1/2\pi = 2.5$ MHz improves the coherence time already by about a factor of 60.

B. Optimal drive parameter in double drive

In principle, we could select for the double drive experiments a very strong first drive Ω_1, but then a stronger second drive Ω_2 is required to correct the noise on the order of $\delta\Omega_1$. Consequently, a balanced first drive Ω_1 (which maximizes Supplementary Fig. 3) will eventually introduce less drive noise Ω_2, and prolong the coherence time of the sensor. For a moderate first drive $\Omega_1/2\pi = 3.363$MHz, the second drive Ω_2 was scanned in Supplementary Fig. 4 in order to find the optimal choice for Ω_2. This scan of Ω_2 has to be done, if the exact magnitude or characteristics of $\delta\Omega_1$ is not known. For each measurement point

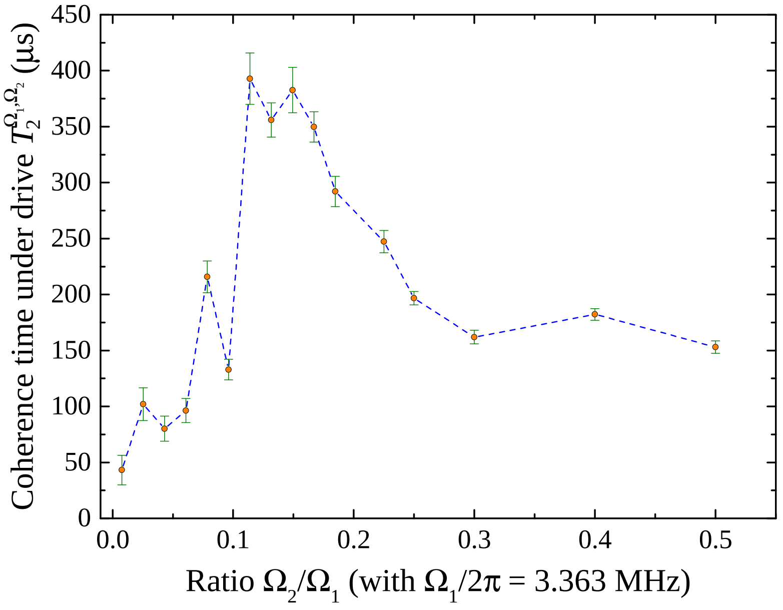

Supplementary Figure 4. **Measurement of the coherence time, $T_2^{\Omega_1,\Omega_2}$, under two drive fields, Ω_1 and Ω_2.** The first drive was set to $\Omega_1/2\pi = 3.363$ MHz and the second drive, Ω_2, is incrementally increased. Since the measurement is sampled at τ_{Ω_1} (see text), we obtain solely a decaying oscillation with $\Omega_2' = \Omega_2/2$, hence, the recorded curve was fitted to $f(t) = c + \alpha \sin\left(\Omega_2' t + \phi\right)\exp(t/T_2^{\Omega_1,\Omega_2})$ to extract the coherence time $T_2^{\Omega_1,\Omega_2}$. The error bars represent the standard deviation $\Delta T_2^{\Omega_1,\Omega_2}$ of the coherence time in the fit. The optimal second drive, Ω_2, maximizes eventually the coherence time $T_2^{\Omega_1,\Omega_2}$ of the sensor.

a decaying trace was recorded, which was (under)sampled at multiples of $\tau_{\Omega_1} = 2\pi/\Omega_1$, i.e. we measure at times $t = N\tau_{\Omega_1}$ ($N \in \mathbb{N}$), which removes the effect of Ω_1 on the recorded traces.

Note that the measured coherence time in Supplementary Fig. 2b is longer compared to the the maximal coherence time obtained in Supplementary Fig. 4. In Supplementary Note 7 it is shown that an applied signal with strength, g, acting partially as a third drive, can further increase the coherence time of the sensor for certain values of g.

Supplementary Note 7: Determine the coherence time of the single drive under an increasing signal

To show the impact of an external signal strength g on the coherence time of the sensor under drive, we perform a single drive measurement (corresponding to one point in Supplementary Fig. 3) by now varying the external signal strength g. We set $\Omega_1/2\pi = 3.002$ MHz and increase gradually g. For each measurement point a decaying trace was recorded, which was (under)sampled at multiples of $\tau_{\Omega_1} = 2\pi/\Omega_1$, i.e. we measure at times $t = N\tau_{\Omega_1}$ ($N \in \mathbb{N}$), which removes the effect of Ω_1 on the recorded traces. Consequently, g' was obtained by fitting the data to an exponential decaying sine function. For small values of g, recording the oscillations of g', and subsequently the coherence time proved to be a challenging measurement, mainly because the period of g' was longer than the coherence time $T_2^{\Omega_1,g}$, so it was difficult to distinguish the two. This caused the measured coherence time to be shorter than expected, given we would expect the coherence time at small g to converge with the measurements without signal (Supplementary Fig. 3), thus having larger error bars, $\Delta T_2^{\Omega_1,g}$, to compensate the ambiguity. Interestingly, the signal acts to a certain extend as a double drive and prolongs the coherence time which is pointed out in Supplementary Note 3. Comparing Supplementary Fig. 4 and Supplementary Fig. 5 two main differences arise.

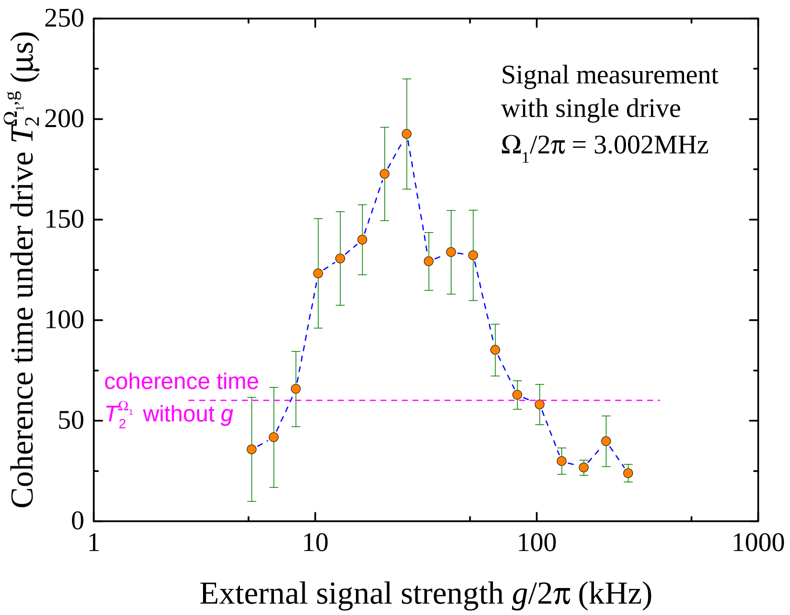

Supplementary Figure 5. Measurement of the coherence time, $T_2^{\Omega_1,g}$, of the qubit for a fixed drive field, Ω_1 and an increasing signal strength, g. The first drive was set to $\Omega_1/2\pi = 3.002$ MHz and an externally applied signal (which is not phase locked to the sensor) is incrementally increased. Since the measurement is sampled at τ_{Ω_1} (see text), we obtain solely a decaying oscillation with $g' = g/2$, hence, the recorded curve was fitted to $f(t) = c + \alpha \sin(g't + \phi)\exp(t/T_2^{\Omega_1,g})$ to extract the coherence time $T_2^{\Omega_1,g}$. The error bars represent the standard deviation $\Delta T_2^{\Omega_1,g}$ of the coherence time in the fit.

First, the maximal coherence times in Supplementary Fig. 5 is reached at a different ratio ($g/\Omega_1 \approx 0.0083$) and second, the maximal coherence time at this point is about the half it was by using Ω_2. This can be explained by the following.

The signal g is not phase looked to the sensor, therefore we would expect that on average $\sum G(t) = \sum g\cos^2(\omega_s t + \langle\varphi\rangle) \approx \frac{1}{2}g$ is contributing to the sensor. A phase locked signal, however, will completely contribute to the decoupling with g, since φ does not vary between each measurement run, which justifies the discrepancy on the y axis in the graphs.

Furthermore, it seems that Ω_2 introduces more noise to the system than g does. As a consequence, $\delta\Omega_2$ is larger and a stronger second drive is needed to cope for the resulting overall noise level.

On the basis of a very rough estimation, we can give some upper limits for the amplitude noise of the microwave fields, which are mentioned in Supplementary Note 4. For the maximal output range of 1 Vpp we can assume 1 Vpp/$2^8 \approx 0.004$ Vpp to be the absolute noise level for this device (Keysight M8195A). Ω_1 was created by 0.75V pp and based on Supplementary Fig. 4 the optimal second drive has to be $\Omega_2 = 0.15\Omega_1 \,\hat{=}\, 0.1125$ Vpp. Eventually, Ω_2 suffers from a larger relative noise level (0.004 Vpp/0.1125 Vpp$\approx$ 3.5 %) then the first drive Ω_1 (0.004 Vpp/0.75 Vpp$\approx$ 0.4%). This statement can be further

confirmed by comparing the relative noise $\delta\Omega_2/\Omega_2$ with $\delta g/g$. The external signal was produced by a microwave generator (R&S SMIQ03B), which runs continuously during the measurements and has a more than 100 times cleaner signal (-70 dBc, relative amplitude noise ratio 0.0003), compared to $\delta\Omega_2/\Omega_2$. This indicates also why the coherence time in Fig. 2 of the main text could be prolonged with g by such a great amount, since the relative (and absolute) noise of the signal g seems to be much smaller than $\delta\Omega_2$. Eventually, a coherence time of 1.5 ms should be almost the limit to which we could extend the coherence time, since the lifetime of the sensor, T_1, was about 3 ms.

Supplementary Note 8: Simulations

In this section we present the results of simulations aimed at reproducing the experimental results and verifying our theoretical model.

A. Magnetic noise

The NV centre spin used in our experiment had a pure dephasing time of $T_2^* = 1.1\,\mu$s, and under a Hahn Echo pulse, a coherence time of $T_2 = 515\,\mu$s. In our model, the magnetic noise has two components. One component, B_r, is a random field that is static within each experiment, but has a different value in different experiments, and a second component, $B(t)$, which is an Ornstein-Uhlenbeck process [6, 7] with a zero expectation value, $\langle B(t)\rangle = 0$, and a correlation function $\left\langle B(t)B\left(t'\right)\right\rangle = \frac{c\tau}{2}e^{-\gamma\left|t-t'\right|}$. B_r is normally distributed with a variance of $\sigma^2 \approx \frac{0.96\times 2}{T_2^*}$, where $T_2^* = 1.1\,\mu$s. An exact simulation algorithm [8] was employed to realize the Ornstein-Uhlenbeck process, which according to

$$B(t+\Delta t) = B(t)e^{-\frac{\Delta t}{\tau}} + n\sqrt{\frac{c\tau}{2}\left(1-e^{-\frac{2\Delta t}{\tau}}\right)}, \tag{25}$$

where n is a unit Gaussian random number. The correlation time of the noise was set to $\tau = 1/\gamma = 10\,\mu$s, where the diffusion constant is given by $c \approx \frac{2}{T_2\tau}$ and corresponds to $S_{BB}(0) \approx \frac{1}{515}$. For pure dephasing, we simulated the Hamiltonian

$$H = \frac{\omega_0}{2}\sigma_z + \left(B_r + B(t)\right)\sigma_z, \tag{26}$$

where we used $\omega_0 = 100$ MHz and the qubit was initialized to $|\uparrow_x\rangle$. The result of the simulation is shown in Supplementary Fig. 6a and corresponds to a pure dephasing time of $T_2^* = 1.1\,\mu$s.

For the Hahn echo pulse we run the same simulation but flipped the sign of $(B_r + B(t))\sigma_z$ at $t = \frac{515}{2}\,\mu$s. The result is shown in Supplementary Fig. 6b and corresponds to a Hahn echo time of $T_2 = 515\,\mu$s.

B. Single drive

We simulated the single-drive scenario under the RWA, so the Hamiltonian is given by

$$H = \frac{\Omega_1}{2}\left(1+\delta_{\Omega_1}(t)\right)\sigma_x + \left(B_r + B(t)\right)\sigma_z. \tag{27}$$

Driving fluctuations were also modelled by an an Ornstein-Uhlenbeck process with a zero expectation value. We chose a correlation time of $\tau_{\Omega_1} = 500\,\mu$s, and a relative amplitude error of $\delta_{\Omega_1} = 0.15\%$ so the diffusion constant is given by $c_{\Omega_1} = 2\delta_{\Omega_1}/\tau_{\Omega}$. We set $\Omega_1 = (2\pi)\cdot 3.4$ MHz, and the qubit was initialized to $|\uparrow_y\rangle$. The result of the simulation is shown in Supplementary Fig. 6c and indicates a coherence time of $T_2^{\Omega_1} \approx 33\,\mu$s, which is in agreement with the experimental results.

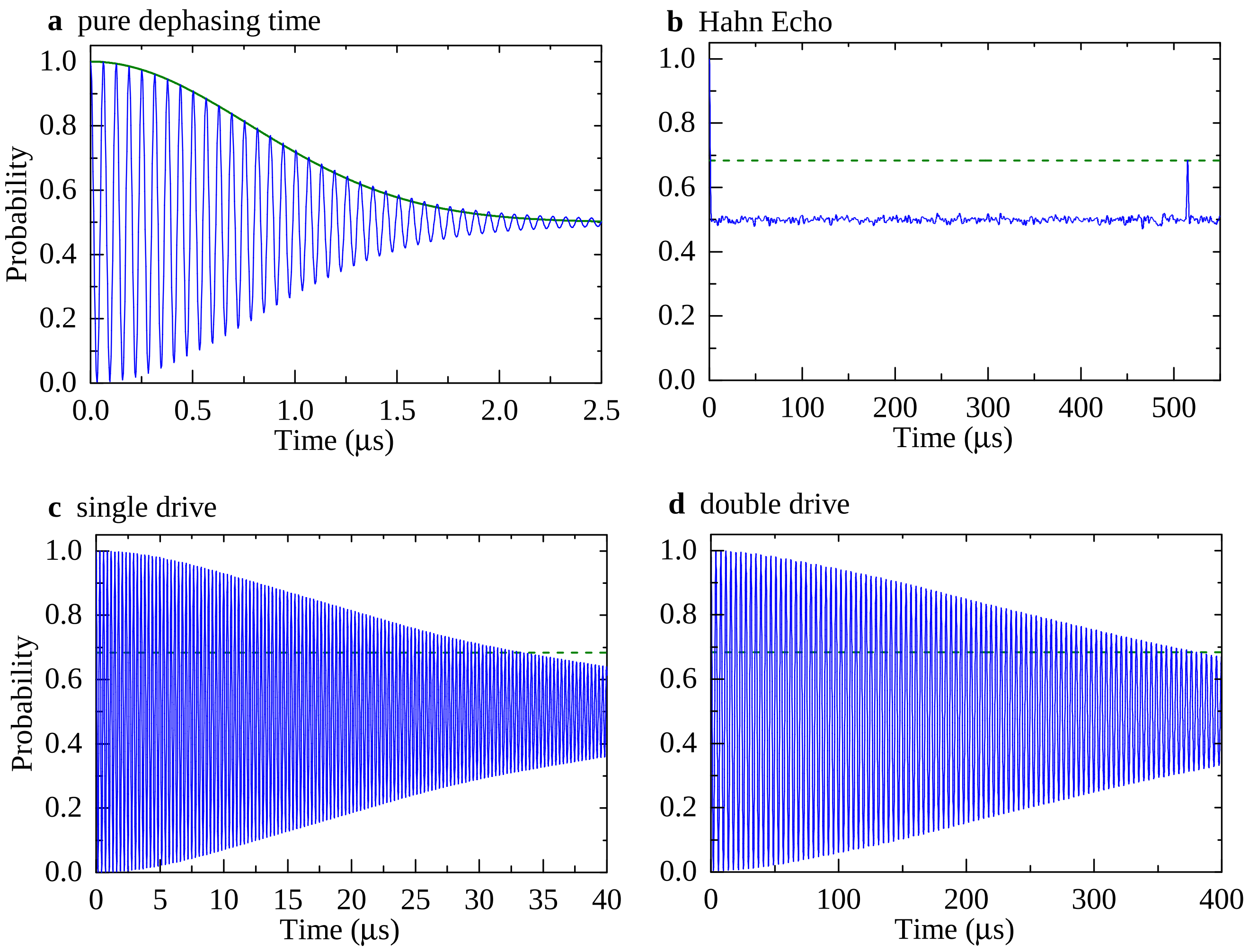

Supplementary Figure 6. **Simulations of coherence times.** (a) Oscillations between $|\uparrow_x\rangle$ and $|\downarrow_x\rangle$ averaged over 5000 trails. $(1+\exp(-\frac{g^2t^2}{2}))/2$ is plotted in green $\left(g^2 = 2/(T_2^*)^2\right)$. (b) Probability of being in the initial $|\uparrow_x\rangle$ state averaged over 2400 trails. Hahn echo pulse at $t = 515/2\,\mu s$ and refocusing at $T_2 = 515\,\mu s$. Dashed horizontal line at $P = (1+1/e)/2$. (c) Coherence time under a single drive with a Rabi frequency of $\Omega_1 = 2\pi \cdot 3.4$ MHz. Oscillations between $|\uparrow_y\rangle$ and $|\downarrow_y\rangle$ averaged over 800 trails. Dashed horizontal line at $P = (1+1/e)/2$. The simulation indicates a coherence time of $T_2^{\Omega_1} \approx 33\,\mu s$. (d) Coherence time under a double drive with Rabi frequencies of $\Omega_1 = 2\pi \cdot 3.4$ MHz and $\Omega_2 = 0.15 \cdot \Omega_1$. Oscillations between $|\uparrow_x\rangle$ and $|\downarrow_x\rangle$ averaged over 800 trails. Dashed horizontal line at $P = (1+1/e)/2$. The simulation indicates a coherence time of $T_2^{\Omega_1\Omega_2} \approx 380\,\mu s$.

C. Double drive

We simulated the double drive scenario in the first IP, making the RWA with respect to ω_0 only. The Hamiltonian is given by

$$H = \frac{\Omega_1}{2}\left(1+\delta_{\Omega_1}(t)\right)\sigma_x + \frac{\Omega_2}{2}\left(1+\delta_{\Omega_2}(t)\right)\cos(\Omega_1 t)\,\sigma_y + \left(B_r + B(t)\right)\sigma_z. \tag{28}$$

Due to some technical issues, in our experimental system the noise in Ω_2 was stronger than the noise in Ω_1. We therefore set $\delta_{\Omega_1} = 0.15\%$ and $\delta_{\Omega_2} = 0.21\%$, where $\Omega_1 = 2\pi \cdot 3.4\,$MHz (like in the single-drive case) and $\Omega_2 = 0.15\Omega_1$. The spin was initialized to $|\uparrow_x\rangle$. The result of the simulation is shown in Supplementary Fig. 6d and indicates a coherence time of $T_2^{\Omega_1,\Omega_2} \approx 380\,\mu s$, which is in agreement with the experimental results.

D. Double drive with a signal

In this section we used the experimental parameters used in the experiment of Supplementary Fig. 2b, where a signal of $g/2\pi = 41.084\,\mathrm{kHz}$ is sensed by the doubly-dressed qubit. The simulation result is shown in Supplementary Fig. 7a and indicates a coherence time of $T_{2,\mathrm{sim}}^{\Omega_1,\Omega_2} \simeq 450\,\mu\mathrm{s}$, which is shorter than the experimental value of $T_{2,\mathrm{exp}}^{\Omega_1,\Omega_2} \simeq 600\,\mu\mathrm{s}$. This could be because our theoretical model of the magnetic noise does not provide a complete characterization of the actual noise, and in fact describes a more severe situation. In principle, the actual spectrum of the noise can be measured [9], which would allow for a theoretical optimization of the parameters used in the sensing protocol. We then varied the value of g in order to verify its effect on the coherence time. For Supplementary Fig. 7b (Supplementary Fig. 7c) we decreased (increased) the value of g by a factor of 2, and used $g/2\pi = 0.5 \cdot 41.084\,\mathrm{kHz}$ ($g/2\pi = 2 \cdot 41.084\,\mathrm{kHz}$). For the smaller (larger) value of g the coherence time was increased (decreased), as expected. We then used the original g value of $g/2\pi = 41.084\,\mathrm{kHz}$, and simulated the improved scheme with $\omega_s = \omega_0 + \frac{\Omega_2}{2}$. The result of this simulation is shown in Supplementary Fig. 7d. The simulation confirms the improved performance of the scheme, and indicates an improvement of the coherence time by ~ 1 order of magnitude.

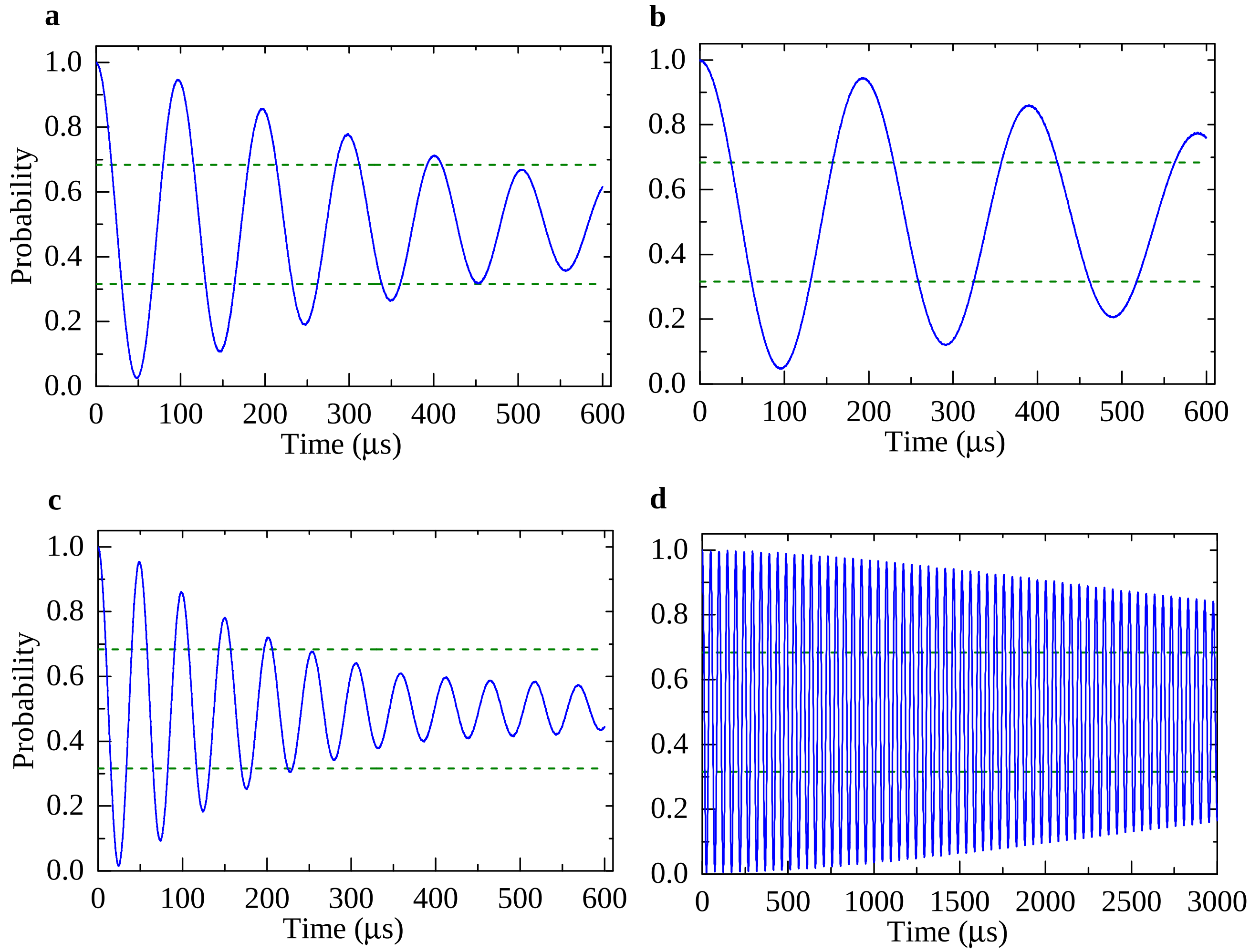

Supplementary Figure 7. **High-frequency signal sensing by a double drive.** Dashed horizontal line at $P = (1 \pm 1/e)/2$. (a) Simulation of high-frequency signal sensing by a double drive with the experimental parameters used in the experiment of Supplementary Fig. 2b. (b) A weaker signal increases the coherence time. All parameters are identical to those of Supplementary Fig. 7a except g, which is decreased by a factor of 2. (c) A stronger signal decreases the coherence time. All parameters are identical to those of Supplementary Fig. 7a except g, which is increased by a factor of 2. (d) Improved scheme. All parameters are identical to those of Supplementary Fig. 7a.

Supplementary Note 9: Pulsed analog of the scheme

The sensing of high frequency fields with a TLS could also be achieved with a pulsed dynamical decoupling analog of our scheme. This could be seen be considering an AC signal with a frequency of $\omega_s = \omega_0 + \Omega$ (similar to the single-drive case). The Hamiltonian of the system under the AC signal is given by

$$H = \frac{\omega_0}{2}\sigma_z + g\sigma_x \cos(\omega_s t), \tag{29}$$

and in the interaction picture with respect to $H_0 = \frac{\omega_0}{2}\sigma_z$ we have that

$$\begin{aligned} H_I &= \frac{g}{2}\left(\sigma_+ e^{-i\Omega t} + \sigma_- e^{+i\Omega t}\right) \\ &= \frac{g}{2}\left(\cos(\Omega t)\,\sigma_x + \sin(\Omega t)\,\sigma_y\right). \end{aligned} \tag{30}$$

Hence, the signal can be measured by initializing the spin to $|\uparrow_x\rangle$ and applying a CPMG pulse sequence, where the pulses correspond to π rotations around the $\hat{y}$ axis (so the σ_y part of the signal is measured). Similar to the common pulsed dynamical decoupling sensing methods, the rate of the pulses should match Ω in order for the signal g to be observed.

Supplementary References

[1] N. Aharon, I. Cohen, F. Jelezko, and A. Retzker, New Journal of Physics **18**, 123012 (2016).

[2] J.-M. Cai, B. Naydenov, R. Pfeiffer, L. P. McGuinness, K. D. Jahnke, F. Jelezko, M. B. Plenio, and A. Retzker, New Journal of Physics **14**, 113023 (2012).

[3] D. F. James and J. Jerke, Canadian Journal of Physics **85**, 625 (2007).

[4] V. Jacques, P. Neumann, J. Beck, M. Markham, D. Twitchen, J. Meijer, F. Kaiser, G. Balasubramanian, F. Jelezko, and J. Wrachtrup, Physical Review Letters **102**, 057403 (2009).

[5] L. M. Pham, *Magnetic Field Sensing with Nitrogen-Vacancy Color Centers in Diamond*, Doctor of Philosophy, Harvard University, Cambridge, Massachusetts (2013).

[6] M. C. Wang and G. E. Uhlenbeck, Reviews of Modern Physics **17**, 323 (1945).

[7] R. Hanson, V. V. Dobrovitski, A. E. Feiguin, O. Gywat, and D. D. Awschalom, Science **320**, 352 (2008).

[8] D. T. Gillespie, Physical Review E **54**, 2084 (1996).

[9] Y. Romach, C. MÃŒller, T. Unden, L. Rogers, T. Isoda, K. Itoh, M. Markham, A. Stacey, J. Meijer, S. Pezzagna, B. Naydenov, L. McGuinness, N. Bar-Gill, and F. Jelezko, Physical Review Letters **114**, 017601 (2015).
